# Supplementary material for: Effects of Austenitizing Temperature on Tensile and Impact Properties of a Martensitic Stainless Steel Containing Metastable Retained Austenite
Source: Materials (Basel). 2021 Feb 20;14(4):1000. doi: 10.3390/ma14041000 (PMC7924182; doi:10.3390/ma14041000)
Supplement: Supplementary file 1 [file materials-14-01000-s001.pdf]

materials

Supplementary Materials

Effects of Austenitizing Temperature on Tensile and Impact Properties of a Martensitic Stainless Steel Containing Metastable Retained Austenite

Biao Deng [1], Dapeng Yang, Guodong Wang, Ziyong Hou and Hongliang Yi

The SADP and EDS are used to distinguish the $M_{23}C_6$ and MC type carbides, as can be seen in Figure S1. The size of the $M_{23}C_6$ carbides is much larger than MC, and the distinction can be used to distinguish the $M_{23}C_6$ and MC in the SEM images.

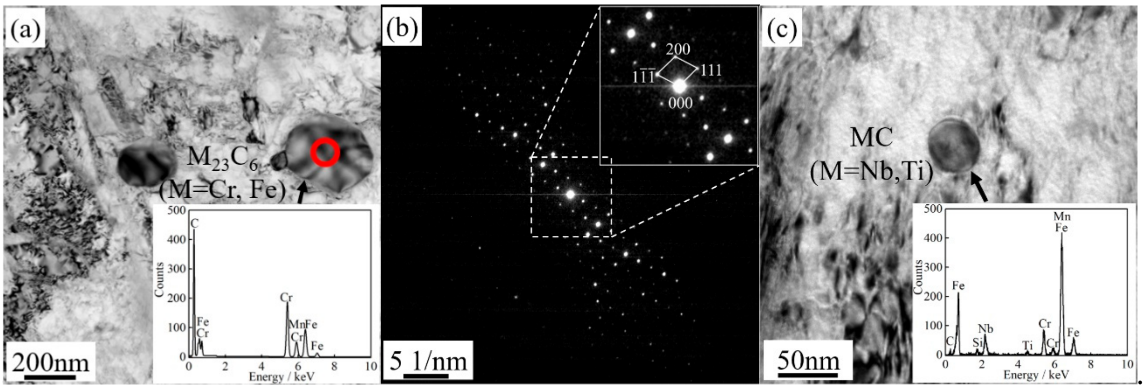

Figure S1. TEM images of (**a**) $M_{23}C_6$ in 1020-Q; (**b**) diffraction patterns of $M_{23}C_6$; (**c**) MC in 1060-Q, where the red annulus represents the selected area of diffraction pattern.

The mole fraction on alloy elements in $M_{23}C_6$ is calculated by Thermo-Calc with the TCFE9 database, as can be seen in Figure S2. The atom ratio of chromium (Cr) and carbon (C) in $M_{23}C_6$ at equilibrium state is approximately 49:20 in the 1000 °C–1040 °C range.

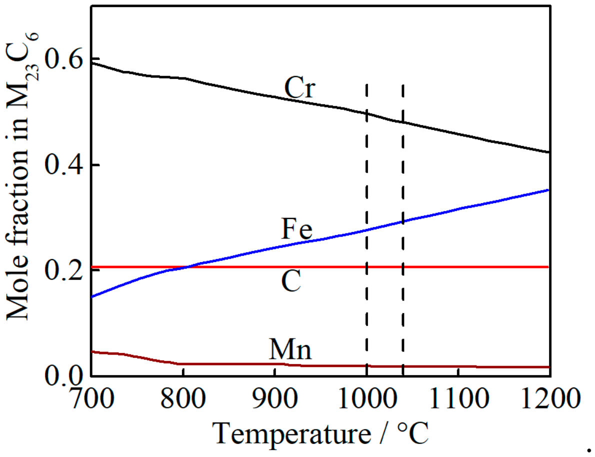

Figure S2. Mole fraction of the alloy elements in $M_{23}C_6$.
